# Tavapadon in Parkinson’s disease: D1/D5 receptor pharmacology, clinical evidence, and translational perspectives

**DOI:** 10.3389/fnagi.2026.1959744

**Published:** 2026-10-12

**Authors:** Mohamed Alsharif, Omran Shrebaty, Mohammad Ammar Aloudah, Raghad Saud AlOubili, Dalal Mohammed Akram Motabagani, Jenan M. Alnamly, Gadi Khalid Sindi, Ahmad Elbana

**Affiliations:** 1 College of Medicine, Alfaisal University, Riyadh, Saudi Arabia; 2 College of Medicine, Sulaiman Al Rajhi University, Al Bukayriyah, Saudi Arabia; 3 College of Medicine, Imam Mohammad Ibn Saud Islamic University (IMSIU), Riyadh, Saudi Arabia; 4 College of Medicine, King Faisal University, Al-Ahsa, Saudi Arabia; 5 College of Medicine, AlMaarefa University, Riyadh, Saudi Arabia; 6 College of Medicine, King Saud Bin Abdulaziz University for Health Sciences, Jeddah, Saudi Arabia; 7 Faculty of Medicine, Sulaiman Al Rajhi University, Al Bukayriyah, Saudi Arabia; 8 Faculty of Medicine, Al-Azhar University, Cairo, Egypt

**Keywords:** D1/D5 receptor, dopamine agonist, levodopa-induced dyskinesia, Parkinson’s disease, partial agonist, tavapadon

## Abstract

Tavapadon is an oral, once-daily, non-catechol partial agonist selective for the D1 and D5 dopamine receptors and represents a mechanistically distinct option currently under clinical investigation for Parkinson disease (PD). This narrative review critically evaluates the biological rationale for D1/D5 receptor targeting, the pharmacological and preclinical development of tavapadon, and the accumulating phase 1–3 clinical evidence for its use as monotherapy in early PD and as adjunctive therapy in patients with motor fluctuations, while highlighting remaining evidence gaps and methodological limitations. A non-systematic literature search of PubMed/MEDLINE, Embase, ClinicalTrials.gov, and Scopus (through May 2026) was performed to identify representative and pivotal randomized controlled trials, pharmacokinetic/pharmacodynamic studies, and mechanistic preclinical investigations relevant to tavapadon and D1/D5 receptor agonism; this narrative approach was not intended to be exhaustive or fully reproducible in the manner of a systematic review. Mechanistically, selective stimulation of the direct striatal pathway together with submaximal intrinsic efficacy at the D1 receptor has been proposed, as a mechanistic hypothesis rather than a demonstrated clinical mechanism, to preserve antiparkinsonian benefit while limiting the receptor-signaling intensity implicated in levodopa-induced dyskinesia. In the phase 2 PARKWAY-II study and the now fully published phase 3 TEMPO-1 and TEMPO-2 trials, fixed- and flexible-dose tavapadon produced clinically meaningful improvements in MDS-UPDRS motor scores compared with placebo in early PD, and in the phase 3 TEMPO-3 trial, adjunctive tavapadon significantly increased daily good ON time and reduced OFF time in levodopa-treated patients with motor fluctuations; efficacy in all trials has been demonstrated only against placebo, with no head-to-head data against levodopa or other dopamine agonists. Adverse events were more frequent with tavapadon than placebo (approximately 72–76% versus 55% across TEMPO-2 and TEMPO-3), with nausea, headache, and dizziness the most common; no excess signal of impulse-control disorders relative to placebo was detected over the available 26–27-week controlled treatment period, and no clinically significant QTc prolongation was observed. The 58-week open-label TEMPO-4 extension, now reported in a peer-reviewed AAN abstract publication in Neurology alongside earlier interim conference data, suggests sustained benefit and a stable long-term levodopa requirement, but lacks a randomized control and cannot yet establish long-term safety superiority over existing agents. Tavapadon is a mechanistically novel candidate with preliminary evidence of efficacy; its ultimate clinical utility will depend on head-to-head trials and long-term safety data.

## Introduction

Parkinson disease (PD) is a progressive neurodegenerative disorder and one of the major causes of neurological disability worldwide (Dorsey et al., 2018; GBD 2016 Parkinson’s Disease Collaborators, 2018). Its clinical diagnosis remains anchored in the motor syndrome of bradykinesia with rigidity, rest tremor, or both, although the disorder extends beyond the nigrostriatal dopaminergic system and includes a broad spectrum of non-motor manifestations (Kalia and Lang, 2015; Poewe et al., 2017). Pathologically, PD is associated with degeneration of dopaminergic neurons in the substantia nigra pars compacta and with alpha-synuclein-rich Lewy pathology, but clinical heterogeneity reflects involvement of multiple neural systems and disease mechanisms (Kalia and Lang, 2015; Spillantini et al., 1997). The rising global burden of PD has intensified the need for therapeutic strategies that are effective, durable, and better tolerated across the disease course (Dorsey et al., 2018; GBD 2016 Parkinson’s Disease Collaborators, 2018).

Levodopa remains the most effective symptomatic therapy for motor manifestations of PD and continues to serve as the benchmark against which emerging dopaminergic therapies are judged (Armstrong and Okun, 2020; Poewe et al., 2017). Chronic levodopa exposure, however, is frequently complicated by wearing-off, motor fluctuations, and levodopa-induced dyskinesia, particularly as disease duration increases (Ahlskog and Muenter, 2001; Espay et al., 2018). Dopamine agonists can reduce early reliance on levodopa and are used as monotherapy or adjunctive therapy, but currently available agents predominantly stimulate D2-like receptors and are limited by adverse effects such as somnolence, hallucinations, orthostatic hypotension, peripheral edema, and impulse-control disorders (Stowe et al., 2008; Voon et al., 2011; Weintraub et al., 2010). In a large cross-sectional study, impulse-control disorders were significantly more common among patients receiving dopamine agonists than among those not receiving them, supporting the clinical relevance of receptor-subtype-related adverse effects (Weintraub et al., 2010). These limitations support continued development of dopaminergic agents with more selective pharmacology and improved benefit-risk profiles (Isaacson et al., 2023).

Dopamine receptors are traditionally divided into D1-like receptors, comprising D1 and D5, and D2-like receptors, comprising D2, D3, and D4 (Beaulieu and Gainetdinov, 2011; Missale et al., 1998). D1 receptors are enriched in striatonigral medium spiny neurons of the direct basal ganglia pathway, where activation promotes movement-facilitating output (Gerfen and Surmeier, 2011; Surmeier et al., 2007). D2 receptors, by contrast, are classically associated with the indirect pathway, and the balance between D1- and D2-mediated signaling is central to basal ganglia models of movement control (Gerfen and Surmeier, 2011; Surmeier et al., 2007). These mechanistic considerations have made selective D1-like receptor stimulation an attractive strategy for PD, particularly because activation of the direct pathway may restore motor function while potentially avoiding adverse effects linked to excessive D2/D3 stimulation (Albin et al., 1989; Isaacson et al., 2023).

The therapeutic pursuit of D1 receptor agonism has been scientifically compelling but historically difficult. Earlier D1 agonists and prodrugs showed antiparkinsonian activity in experimental and clinical settings, but their development was limited by short duration of action, poor oral bioavailability, cardiovascular adverse effects, and tolerability problems (Albin et al., 1989; Blanchet et al., 1998; Rascol et al., 1999). These limitations created a need for non-catechol, orally bioavailable D1/D5-selective compounds with pharmacokinetic properties suitable for chronic PD treatment (Hall et al., 2019; Isaacson et al., 2023).

Tavapadon, formerly PF-06649751 and CVL-751, is an oral, once-daily, selective D1/D5 receptor partial agonist developed to address this therapeutic gap (Bezard et al., 2024; Hall et al., 2019; Sohur et al., 2018). Its partial agonist profile is intended to provide sufficient D1/D5 receptor activation for motor benefit while avoiding excessive receptor stimulation, a rationale distinct from both levodopa and conventional D2/D3-preferring dopamine agonists (Hall et al., 2019; Isaacson et al., 2023). Phase 1 studies in patients with PD showed that PF-06649751 was generally safe and well tolerated, with pharmacokinetic and pharmacodynamic findings supporting further evaluation (Sohur et al., 2018). A subsequent randomized, placebo-controlled phase 2 study in early PD reported significant improvement in MDS-UPDRS Part III motor scores despite early termination for reasons unrelated to safety (Riesenberg et al., 2020).

The clinical evidence base for tavapadon has since expanded with phase 3 testing. In the TEMPO-1 randomized clinical trial, fixed-dose tavapadon improved motor outcomes in early PD compared with placebo over 26 weeks (Pahwa et al., 2026). In the TEMPO-3 randomized clinical trial, adjunctive tavapadon in levodopa-treated patients with motor fluctuations significantly increased daily good ON time and reduced OFF time compared with placebo (Fernandez et al., 2026a). These findings, taken as a set, point to selective D1/D5 partial agonism being relevant both as early symptomatic monotherapy and as an adjunctive strategy in fluctuating PD (Fernandez et al., 2026a; Pahwa et al., 2026). Important questions nonetheless remain regarding long-term durability, comparative effectiveness against existing dopaminergic strategies, neuropsychiatric safety, dyskinesia risk, and optimal positioning within treatment algorithms (Hall et al., 2019; Isaacson et al., 2023; Pahwa et al., 2026).

This narrative review summarizes the biological rationale for D1/D5 receptor targeting in PD, traces the pharmacological development of tavapadon, critically evaluates the available clinical evidence, and considers how selective D1/D5 partial agonism may influence future PD pharmacotherapy.

## Method

This is a narrative review, where a non-exhaustive literature search was conducted across four electronic databases, PubMed/MEDLINE, Embase, ClinicalTrials.gov, and Scopus, with a date limit extending to May 2026, to identify representative and pivotal literature on tavapadon rather than to achieve complete, reproducible study identification. Search terms combined (“tavapadon” OR “PF-06649751” OR “CVL-751”) AND (“Parkinson’s disease” OR “motor fluctuations”) AND (“D1 receptor” OR “dopamine agonist”). Eligible sources were phase 1–3 randomized controlled trials, pharmacokinetic or pharmacodynamic characterization studies, and mechanistic preclinical investigations published in English in peer-reviewed journals; conference abstracts and trial-registry records were used only where no peer-reviewed publication was yet available (as for interim TEMPO-4 safety data at the time of writing) and are identified as such wherever cited, with correspondingly reduced evidentiary weight, rather than being treated as equivalent to full peer-reviewed reports. Because study selection was not conducted as a formal, reproducible systematic process, no record-count flow diagram is reported; a single reviewer identified and the senior author confirmed the sources synthesized below. Given the heterogeneity of study designs across phase 1 pharmacokinetic/pharmacodynamic work, phase 2/3 randomized trials, and preclinical primate models, findings were synthesized narratively and thematically across the domains of pharmacology, preclinical efficacy, clinical efficacy, and safety, rather than pooled quantitatively. Critical appraisal emphasized trial design, statistical power, comparator choice, and generalizability.

## D1/D5 receptor pharmacology

Dopamine receptors are G protein-coupled receptors classified into two pharmacologically and structurally distinct families based on preferential G protein coupling. The D1-class receptors, comprising D1 and D5 subtypes, couple canonically to G-alpha-s and the closely related striatum-enriched G-alpha-olf, both of which stimulate adenylyl cyclase activity. The D2-class receptors, comprising D2, D3, and D4 subtypes, couple primarily to G-alpha-i/o proteins, which inhibit adenylyl cyclase. D1 and D5 receptors are frequently treated as pharmacologically interchangeable given their high sequence homology and the lack of subtype-selective ligands, yet the D5 receptor has a more restricted central expression pattern and has been implicated in functions distinct from the D1 receptor, including modulation of cholinergic striatal interneurons during levodopa-induced dyskinesia (Jones-Tabah et al., 2022). The D5 receptor in particular is enriched in striatal cholinergic interneurons, where its activation shapes cortical oscillations and cognitive processing, an attribute that may differentiate tavapadon from pure D1 agonists and that carries distinct implications for non-motor symptoms and dyskinesia modulation (Church, 2021; Li et al., 2025).

D1-class receptor activation stimulates adenylyl cyclase via G-alpha-s/olf, raising intracellular cyclic adenosine monophosphate and activating protein kinase A, which then phosphorylates substrates throughout the cytosol, synaptic membrane, and nucleus. In the striatum, this cascade converges heavily on dopamine- and cAMP-regulated phosphoprotein of 32 kDa (DARPP-32), which amplifies protein kinase A signaling by inhibiting protein phosphatase-1, generating a positive feedback loop that is particularly pronounced in striatal medium spiny neurons relative to other neuronal populations. Striatal neurons respond to brief, sub-second pulses of dopamine with strong and long-lasting cAMP/protein kinase A activation, a property not shared by cortical neurons, which instead require sustained receptor stimulation to produce comparable signaling. D2-class receptor signaling has the pharmacologically opposite effect, inhibiting adenylyl cyclase and reducing cAMP/protein kinase A activity, so that dopamine exerts antagonistic intracellular effects depending on which receptor class predominates in a given neuronal population (Jones-Tabah et al., 2022).

Molecular evidence for the functional segregation of striatal output pathways came from Gerfen and colleagues, who showed that striatonigral neurons projecting through the direct pathway selectively express D1 receptors together with the neuropeptides substance P and dynorphin, whereas striatopallidal neurons projecting through the indirect pathway selectively express D2 receptors together with enkephalin (Gerfen et al., 1990). Using a 6-hydroxydopamine rat lesion model, the same study found that loss of striatal dopamine produces reciprocal changes in receptor and neuropeptide gene expression across these two populations (Gerfen et al., 1990). Because D1 receptors couple to stimulatory G-alpha-s/olf proteins and D2 receptors couple to inhibitory G-alpha-i/o proteins (Jones-Tabah et al., 2022), dopamine exerts pharmacologically opposite effects on the two pathway populations despite arising from the same nigrostriatal projection. Figure 1 summarizes this anatomical and pharmacological segregation and situates tavapadon’s proposed mechanism of action within it.

**FIGURE 1:**
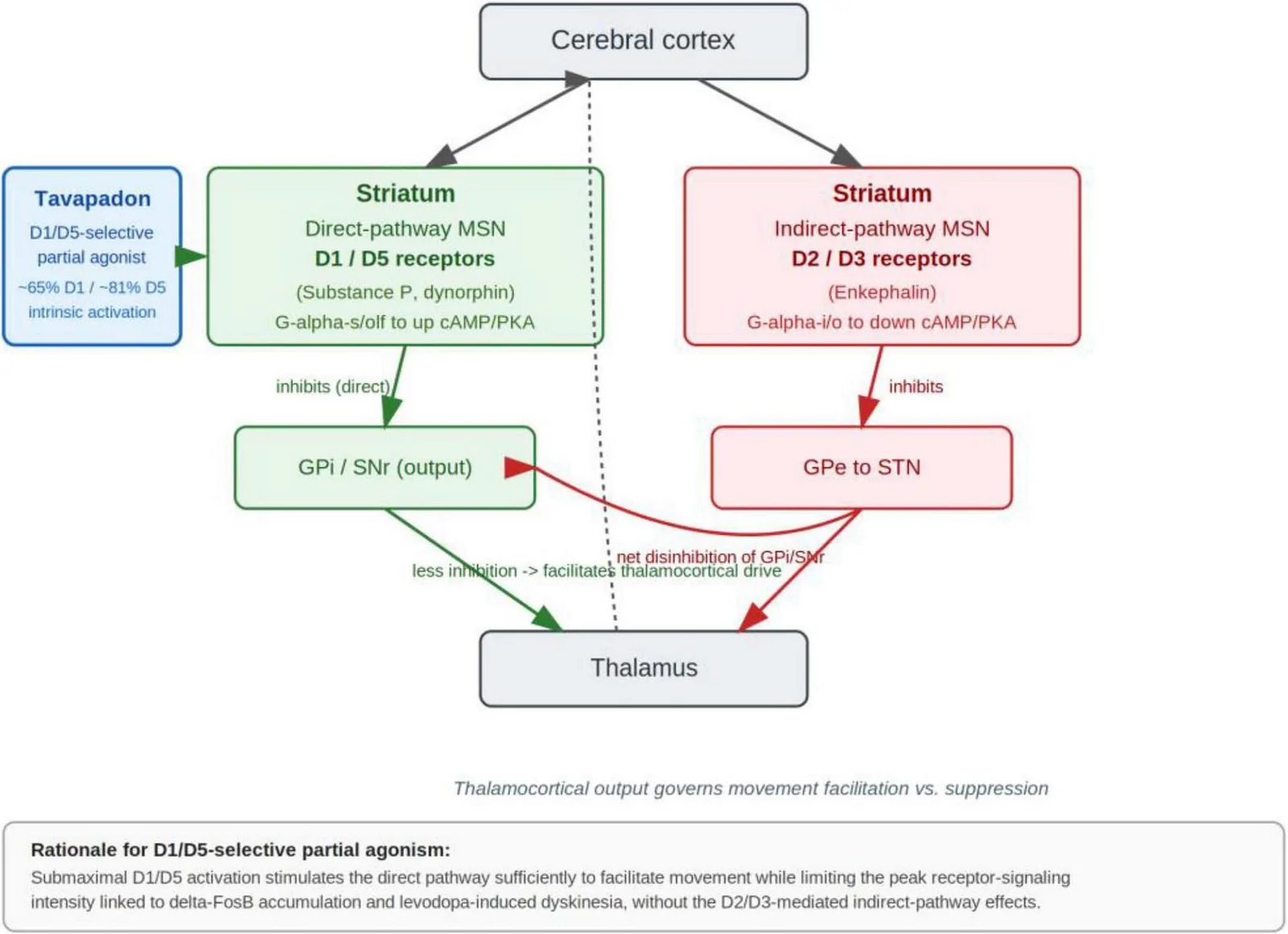
Schematic of the direct (D1/D5) and indirect (D2/D3) striatal output pathways and the proposed mechanism of tavapadon. Cortical glutamatergic input drives two pharmacologically opposed medium spiny neuron (MSN) populations. Direct-pathway MSNs are principally characterized by D1 receptor expression coupled to stimulatory G-alpha-s/olf signaling and inhibit GPi/SNr output, disinhibiting the thalamus and facilitating movement; D5 receptor expression is more heterogeneous and is not itself a defining marker of this pathway. Indirect-pathway MSNs express D2/D3 receptors coupled to inhibitory G-alpha-i/o signaling and, via the GPe-STN relay, drive net disinhibition of GPi/SNr, suppressing thalamocortical output; contemporary circuit models indicate that direct- and indirect-pathway MSNs are frequently co-activated during movement rather than acting in strict reciprocal opposition, so this diagram is a simplified schematic rather than a literal description of basal ganglia dynamics. Tavapadon acts as a selective partial agonist at D1/D5 receptors, with its direct-pathway effects mediated predominantly through D1 (approximately 65% intrinsic activation at D1 and 81% at D5 in *in vitro* cAMP/functional assays relative to a full agonist, not receptor occupancy or a percentage of clinical receptor activation), engaging the direct pathway with submaximal signaling intensity, a profile proposed to preserve antiparkinsonian benefit while limiting the peak D1-coupled signaling associated with delta-FosB accumulation and levodopa-induced dyskinesia. MSN, medium spiny neuron; GPi, globus pallidus interna; GPe, globus pallidus externa; SNr, substantia nigra pars reticulata; STN, subthalamic nucleus; PKA, protein kinase A.

More recent systems-neuroscience evidence complicates the strict antagonism implied by the classic rate model. Calabresi and colleagues reviewed *in vivo* recording and optogenetic studies showing that direct- and indirect-pathway medium spiny neurons are frequently co-activated during normal movement, and proposed that the functional output of each population is shaped less by pathway identity than by the form of synaptic plasticity expressed at corticostriatal synapses at a given moment (Calabresi et al., 2014). This reappraisal tempers the assumption that direct- and indirect-pathway activity are simply reciprocal, with direct implications for how selective D1/D5 receptor stimulation should be expected to influence motor output in the dopamine-depleted striatum.

A central pharmacological liability of D2/D3 receptor agonists used in PD is that, by suppressing indirect-pathway output beyond the physiological range achieved by endogenous dopaminergic tone, they risk overcorrecting basal ganglia circuitry. Within the rate-model framework, indirect-pathway suppression beyond what is required to restore normal globus pallidus interna/substantia nigra pars reticulata activity shifts basal ganglia output toward a pro-dyskinetic state (Albin et al., 1989). Beyond dyskinesia, D2/D3 agonists are mechanistically linked to dose-limiting adverse effects including impulse-control disorders, excessive daytime sleepiness, and psychosis, thought to arise from off-target activation of D2/D3 receptors in the mesolimbic pathway rather than from direct-pathway D1 activity. Pramipexole and ropinirole, which have the highest D3 selectivity among approved agonists, carry the highest rates of impulse-control disorders, consistent with a mesolimbic D3-mediated mechanism (Isaacson et al., 2023).

Independent of indirect-pathway overcorrection, a second, D1-specific mechanism contributes to dyskinesia and provides the core rationale for D1/D5-selective partial agonism. Aubert and colleagues, using a non-human primate model of levodopa-induced dyskinesia, found that striatal D1 receptor density itself was not increased in dyskinetic animals, but that D1 receptor-coupled signal transduction was markedly sensitized, and that this sensitization correlated linearly with dyskinesia severity; the supersensitivity was accompanied by elevated striatal levels of cyclin-dependent kinase 5 and DARPP-32, both downstream effectors of D1-coupled cAMP/protein kinase A signaling (Aubert et al., 2005).

Subsequent work has converged on the transcription factor delta-FosB as a key molecular substrate linking direct-pathway D1 receptor overactivation to levodopa-induced dyskinesia. Using the MPTP non-human primate model of parkinsonism, Calon and colleagues showed that dyskinesia-inducing dopaminergic regimens, whether levodopa or selective D1 or D2 agonists, induced accumulation of chronic delta-FosB-family proteins in the striatum when the drugs were short-acting and administered in a pulsatile mode; these proteins combine with Jun-D to form AP-1 transcriptional complexes capable of altering expression of genes including enkephalin and the N-methyl-D-aspartate receptor. The authors proposed that levodopa-induced dyskinesia represents a form of pathological synaptic learning arising from deficient gating of glutamatergic corticostriatal input by dopamine. Both D1 and D2 agonists were each capable of inducing dyskinesia when given in a pulsatile, short-acting fashion. This points to pulsatility and intensity of receptor stimulation, rather than receptor identity alone, as a key driver of dyskinesia priming (Calon et al., 2000).

Using D1-Cre transgenic rats and *in vivo* fiber photometry, Gao and colleagues provided direct, real-time evidence for this model, demonstrating aberrant, exaggerated activation of striatal D1 receptor-positive medium spiny neurons during dyskinetic episodes following chronic levodopa treatment; chemogenetic inhibition of this neuronal population delayed the onset of dyskinetic behavior (Gao et al., 2022). Jones-Tabah and colleagues further note that this D1-mediated sensitization specifically engages downstream ERK1/2 and mTORC1 signaling and chromatin-level changes such as histone H3 phosphorylation at the Fosb promoter, and that interventions disrupting these downstream nodes, rather than D1 receptor activation itself, can attenuate dyskinesia without abolishing the therapeutic motor benefit of dopaminergic stimulation (Jones-Tabah et al., 2022).

Two arguments for D1/D5-selective partial agonism emerge from this body of work. First, because the indirect pathway is D2-mediated and anatomically and molecularly distinct from the direct pathway (Gerfen et al., 1990), an agent acting selectively at D1/D5 receptors should, in principle, restore direct-pathway facilitation of movement without the supraphysiological indirect-pathway suppression associated with D2/D3 agonists (Albin et al., 1989). Second, because the dyskinesia-promoting substrate appears to depend specifically on the degree of D1 receptor signaling intensity rather than receptor expression or engagement *per se* (Aubert et al., 2005), and on the temporal pattern of that stimulation, with pulsatile administration a key driver of delta-FosB accumulation regardless of receptor class (Calon et al., 2000), a partial agonist with submaximal intrinsic efficacy at the D1 receptor may limit the peak signaling amplitude responsible for delta-FosB accumulation and dyskinesia priming. This remains a mechanistic hypothesis rather than a demonstrated basis for tavapadon’s clinical effects: receptor occupancy and intrinsic efficacy, downstream signaling bias, disease stage and prior dopaminergic exposure, and network-level striatal plasticity are all additional determinants of dyskinesia risk that have not been isolated in tavapadon-specific studies, and the phase 3 trials to date establish symptomatic efficacy and short-term safety rather than a disease-modifying or dyskinesia-preventive effect in humans.

Despite this mechanistic rationale, early attempts to develop clinically viable full D1 agonists were largely unsuccessful. Dihydrexidine, the first full D1/D5 agonist to be pharmacologically characterized, demonstrated antiparkinsonian efficacy in a primate model of PD and produced measurable, if brief, motor improvement in a small placebo-controlled human trial. In that trial, the only patient to achieve a plasma concentration above 100 ng/mL showed transient motor improvement accompanied by choreic dyskinesia resembling the levodopa response, but dose-limiting adverse effects, including flushing, hypotension, and tachycardia, occurred in all patients, particularly with rapid intravenous infusion, and the plasma half-life was under 5 min. These pharmacokinetic and cardiovascular liabilities, rather than any failure of the underlying D1-selectivity hypothesis, were judged to severely limit the compound’s therapeutic window (Blanchet et al., 1998).

Dinapsoline, another full D1 agonist characterized in the early 2000s, showed robust efficacy in a 6-hydroxydopamine-lesioned rat model of PD, producing marked contralateral rotational behavior after subcutaneous or oral administration, an effect attenuated by the D1 antagonist SCH-23390 but not by the D2 antagonist raclopride, confirming D1-mediated action. Unlike some earlier D1 agonists, dinapsoline did not produce behavioral tolerance over a 14-day chronic dosing period. However, its relatively limited D1:D2 selectivity *in vitro*, combined with pharmacokinetic and tolerability constraints typical of catechol-based D1 agonists of that era, similarly precluded clinical development (Gulwadi et al., 2001). The failure of this generation of compounds reflected the unavoidable pharmacokinetic and cardiovascular liabilities of the catechol pharmacophore rather than any deficiency in the D1-selectivity hypothesis, and this precedent is the explicit justification for the non-catechol scaffold of tavapadon.

Reviewing this generation of compounds, Jones-Tabah and colleagues observe that essentially all early selective D1/D5 receptor agonists contained a catechol (dihydroxyphenyl) chemical group, a moiety that limits oral bioavailability and central nervous system penetration and renders these compounds susceptible to rapid metabolism; this chemistry-related limitation, rather than any deficiency in the D1-selective mechanistic rationale, is identified as the principal reason clinical development of D1/D5 agonists was abandoned for an extended period. The authors further note that despite nearly four decades of investigation, no centrally acting D1/D5 receptor ligand had reached clinical use prior to the recent emergence of non-catecholamine chemical scaffolds with improved pharmacokinetic profiles and reduced receptor desensitization (Jones-Tabah et al., 2022). This arc, robust proof-of-mechanism efficacy paired with prohibitive chemistry-driven pharmacokinetic and tolerability failures, constitutes the direct rationale for the partial-agonist, non-catechol design strategy later adopted in the development of tavapadon.

## Tavapadon: pharmacology and preclinical profile

Dopaminergic agonists act on different dopamine receptor subtypes and exert their clinical effects accordingly. Tavapadon is a selective D1 and D5 receptor partial agonist that stimulates the direct striatal pathway in the basal ganglia circuit (Bezard et al., 2024). D1 and D5 receptors show the highest affinity for tavapadon (Ki of approximately 9 nM and 13 nM, respectively) relative to D2, D3, and D4 receptors (Ki of at least 6210 nM, 6720 nM, and 4870 nM, respectively) (Bezard et al., 2024; Kaye et al., 2025). Because of its partial agonism, tavapadon activates the D1 receptor by approximately 65% and the D5 receptor by approximately 81% (Bezard et al., 2024); this partial activation may help relieve the motor symptoms of PD while theoretically minimizing adverse events such as dyskinesia (Kaye et al., 2025). Tavapadon is given orally, is rapidly absorbed, and has a terminal half-life of approximately 20–24 h, allowing once-daily dosing. This longer half-life may support better long-term adherence and symptom control than levodopa, whose short half-life of approximately 1–2 h contributes to fluctuating plasma dopamine levels and the ON/OFF phenomenon that prompts multiple daily doses. Tavapadon is cleared mainly through cytochrome P450 3A4 (CYP3A4) (Kaye et al., 2025; Pastino et al., 2022), and consequently interacts with drugs that share this metabolic pathway: CYP3A4 inducers may lower tavapadon plasma concentrations, while CYP3A4 inhibitors may raise them. Co-administration with itraconazole, a strong CYP3A4 inhibitor, has been shown to raise tavapadon peak concentration and overall exposure by approximately fourfold and fivefold, respectively (Pastino et al., 2022); concomitant use with other strong CYP3A4 inhibitors (for example, clarithromycin or ritonavir) or inducers (for example, rifampicin or phenytoin) would therefore be expected to require dose adjustment or avoidance, although specific guidelines have not yet been established. Levodopa, unlike tavapadon, is metabolized peripherally by amino acid decarboxylase, catechol-O-methyltransferase, and monoamine oxidase; tavapadon’s independence from these pathways may support more sustained control of motor fluctuations than the pulsatile dopaminergic exposure typical of standard agents (Kaye et al., 2025; Pastino et al., 2022).

Preclinical studies in monkeys and rodents have compared tavapadon with levodopa (Bezard et al., 2024). In one study, macaques were rendered parkinsonian with MPTP and then treated with levodopa to induce levodopa-induced dyskinesia, after which three experiments assessed the efficacy and safety of CVL-751 relative to levodopa. The first compared tavapadon and levodopa as separate groups and found that tavapadon at 0.04 mg/kg and full-dose levodopa produced comparable improvements in disability score and locomotor function, with markedly less dyskinesia in the tavapadon group. The second combined levodopa and tavapadon at multiple doses; the most clinically relevant combination, 33% of the standard levodopa dose with tavapadon at 0.04 mg/kg, reduced the disability score as effectively as full-dose levodopa while still meaningfully reducing dyskinesia, suggesting a preclinical pharmacodynamic interaction consistent with a possible levodopa-sparing effect; this is hypothesis-generating and preclinical only, and should not be extrapolated to a specific magnitude of levodopa dose reduction in human patients, which has not been clinically demonstrated. The third assessed higher tavapadon doses (0.10 and 0.15 mg/kg) and found similar efficacy along with longer symptom relief than levodopa (350 min versus 120 min). Tavapadon, unlike most dopaminergic agents, did not show a dose-dependent rise in dyskinesia risk as the dose was escalated (Young et al., 2020). These primate findings are encouraging, but the MPTP model primarily reflects acute dopaminergic depletion rather than the progressive, multi-neurotransmitter degeneration seen in human PD, which may overestimate the translational durability of the effect.

## Clinical evidence

The efficacy and safety of tavapadon have been examined in several recent clinical trials spanning early and fluctuating PD, evaluating its use both as monotherapy and as adjunctive treatment to standard dopaminergic therapy (ClinicalTrials.gov, 2026a; Fernandez et al., 2026a,b; Pahwa et al., 2026; Riesenberg et al., 2020). The PARKWAY-II trial, a 15-week, phase 2, double-blind, randomized study, compared tavapadon (PF-06649751) monotherapy with placebo in 57 patients with early PD, with the primary endpoint being mean change from baseline in Movement Disorder Society-Unified Parkinson’s Disease Rating Scale (MDS-UPDRS) Part III score. Patients began on a minimum dose of 0.25 mg, underwent mandatory escalation to 0.75 mg, and were then titrated up to a maximum of 15 mg at the investigator’s discretion to balance symptom relief and tolerability. Tavapadon was well tolerated during the maintenance phase, in which 11 of 29 patients received 15 mg and four received 5 mg. Relative to placebo, the tavapadon group showed a 4.8-point reduction from baseline at week 15 (*p* = 0.0407), with reductions also evident at weeks 3, 5, 7, 9, and 12, suggesting a sustained effect throughout the study. PARKWAY-II concluded that tavapadon improved motor symptoms and was generally well tolerated in early PD, although the small sample size of 57 patients limits generalizability to a more diverse population (Riesenberg et al., 2020).

The four TEMPO trials form the core of the phase 3 program evaluating tavapadon’s efficacy, safety, and tolerability. TEMPO-1 and TEMPO-2 were phase 3 trials using fixed-dose and flexible-dose regimens, respectively, to assess tavapadon monotherapy, while TEMPO-3 evaluated tavapadon as adjunctive treatment to levodopa (ClinicalTrials.gov, 2026a; Fernandez et al., 2026a,b; Pahwa et al., 2026). Adults with early PD were enrolled in TEMPO-1 and TEMPO-2, with the primary outcome being mean change from baseline to week 26 in combined MDS-UPDRS Part II and III scores (ClinicalTrials.gov, 2026a; Fernandez et al., 2026b; Pahwa et al., 2026). Table 1 summarizes the design and primary outcome of each of these trials alongside PARKWAY-II.

**TABLE 1:** Summary of key tavapadon clinical trials (ClinicalTrials.gov, 2026a; Fernandez et al., 2026a; Pahwa et al., 2026; Riesenberg et al., 2020).

Trial	Phase	Population	Regimen	Duration	Primary endpoint	Tavapadon result (treatment diff. vs. placebo, 95% CI)	Placebo result	*P*-value	Source
PARKWAY-II	2	Early PD (*n* = 57)	Flexible 0.25–15 mg	15 wk	Change in MDS-UPDRS III	−4.8 (95% CI not reported)	∼0	0.0407	Riesenberg et al., 2020
TEMPO-1	3	Early PD (*n* = 529)	Fixed 5 mg / 15 mg	26 wk	Change in MDS-UPDRS II+III	Diff. –11.5 (95% CI −13.8, −9.2) / −12.1 (−14.4, −9.8)	+1.8	< 0.001	Pahwa et al., 2026
TEMPO-2	3	Early PD (*n* = 304)	Flexible 5–15 mg	26 wk	Change in MDS-UPDRS II+III	−10.3; Diff. −9.1 (95% CI −11.7, −6.5)	−1.2	< 0.0001	Fernandez et al., 2026b
TEMPO-3	3	Fluctuating PD (*n* = 507)	Adjunct to levodopa	26 wk	Change in daily good ON time	+1.70 h; Diff. +1.10 h (95% CI 0.60, 1.70)	+0.6 h	< 0.001	Fernandez et al., 2026a

TEMPO-1, a multicenter, double-blind, placebo-controlled trial, randomized 529 patients 1:1:1 to placebo, tavapadon 5 mg, or tavapadon 15 mg. Both tavapadon groups showed pronounced improvement from baseline to week 26 in combined MDS-UPDRS scores relative to placebo (a decrease of 9.7 points and 10.2 points, respectively, versus a 1.8-point increase with placebo), a treatment difference of nearly 12 points (Pahwa et al., 2026). TEMPO-2, also randomized and placebo-controlled and now fully reported in a peer-reviewed publication, allocated 304 patients 1:1 to placebo or flexible-dose tavapadon (5–15 mg), and found a substantially greater reduction in combined MDS-UPDRS Part II and III scores with tavapadon than placebo (10.3-point versus 1.2-point decrease; treatment difference −9.1 points, 95% CI −11.7 to −6.5, *p* < 0.0001); adverse events (76% versus 55%) and discontinuations (38% versus 15%) were both more frequent with tavapadon than placebo, mainly during dose titration (Fernandez et al., 2026b). TEMPO-1 and TEMPO-2, taken together, support a beneficial role for tavapadon versus placebo in reducing motor symptoms in early PD, though neither establishes superiority over levodopa or other dopamine agonists in the absence of head-to-head trials (ClinicalTrials.gov, 2026a; Fernandez et al., 2026b; Pahwa et al., 2026). TEMPO-3 evaluated tavapadon as adjunctive therapy in 507 patients with motor fluctuations who were maintained on at least 400 mg/day of levodopa; the primary endpoint was change from baseline to week 26 in total daily ON time without troublesome dyskinesia. Daily good ON time increased by 1.7 h with tavapadon compared with 0.6 h with placebo, supporting efficacy in improving daily ON time, although the authors noted that larger, longer-term studies are needed (Fernandez et al., 2026a). Across the TEMPO program, then, tavapadon has demonstrated efficacy versus placebo across different stages of PD, whether given as monotherapy or added to levodopa, though this does not establish superiority over levodopa, pramipexole, ropinirole, rotigotine, or other current strategies for early PD or motor fluctuations, which have not been directly compared with tavapadon (ClinicalTrials.gov, 2026a; Fernandez et al., 2026a,b; Pahwa et al., 2026). The TEMPO-4 trial assessed the long-term effects of tavapadon over 58 weeks in an open-label extension enrolling 992 patients previously included in the TEMPO program, who received either 5 mg or 15 mg of tavapadon; the study monitored adverse events including changes in electrocardiogram findings, laboratory values, and vital signs. Levodopa-use outcomes from TEMPO-4 have since been reported in a peer-reviewed AAN abstract publication in Neurology, indicating that approximately 86.4–94.0% of participants entering from the early-PD TEMPO-1/TEMPO-2 cohorts avoided levodopa initiation over 58–85 weeks of tavapadon treatment, and that 81.3–87.6% of participants already receiving levodopa (from TEMPO-3 or newly enrolled) maintained a stable dose, with dose reductions more common than increases; broader safety and efficacy findings from TEMPO-4 have also been reported in interim form at the 2025 MDS Congress. These are open-label, uncontrolled findings without a randomized comparator, so they should be interpreted as preliminary evidence of durability and levodopa-sparing potential rather than confirmed long-term efficacy or safety, and the entire evidence base can no longer be described as consisting solely of unpublished data (ClinicalTrials.gov, 2026b; Ondo et al., 2026).

Non-motor outcomes were not primary or secondary endpoints in any of the trials described above but were assessed as safety measures. TEMPO-1, TEMPO-3, and the phase 2 PARKWAY trial measured sleepiness, impulse control, and suicidal risk using the Epworth Sleepiness Scale, the Questionnaire for Impulsive-Compulsive Disorders in Parkinson’s Disease-Rating Scale, and the Columbia-Suicide Severity Rating Scale, respectively. Across these studies, no statistically significant difference was found between tavapadon and placebo on any of these three measures, suggesting no excess signal of these adverse effects was detected during the available controlled treatment period, though the duration and sample size are insufficient to exclude a clinically meaningful difference over longer exposure (ClinicalTrials.gov, 2026a; Fernandez et al., 2026b; Pahwa et al., 2026; Riesenberg et al., 2020).

## Safety and tolerability

Parkinson’s disease treatment has long been dominated by levodopa and D2/D3-selective dopamine agonists, both of which are often limited by motor fluctuations and a substantial risk of adverse effects (Bezard et al., 2024). Tavapadon represents a different approach: a highly selective partial agonist at D1 and D5 dopamine receptors that targets the direct motor pathway to facilitate movement while avoiding the broad activation of the indirect pathway associated with D2/D3 receptors (Bezard et al., 2024). Unlike traditional catechol-based agonists, tavapadon is G protein-biased, meaning it preferentially promotes stimulatory signaling while reducing beta-arrestin recruitment, which may limit receptor desensitization and behavioral tolerance (Shi et al., 2025). Its action at the D5 receptor is also of interest, given that these receptors are enriched in cholinergic interneurons that influence both motor output and cognitive oscillations (Church, 2021; Li et al., 2025).

No excess safety signal has emerged to date for tavapadon relative to placebo over the available 26–27-week controlled trial periods, though adverse events overall were more common with tavapadon than placebo across TEMPO-2 (76% versus 55%) and TEMPO-3 (72% versus 55%), with nausea, headache, and dizziness the most frequently reported adverse events, and discontinuation due to adverse events more frequent with tavapadon (Kaye et al., 2025). Nausea and vomiting are dose-dependent, typically occur during up-titration, and tend to resolve with continued exposure (Fernandez et al., 2026a; Pahwa et al., 2026), a pattern consistent across early-stage and advanced PD populations (Pahwa et al., 2026; Pringsheim et al., 2021). Regarding orthostatic hypotension, tavapadon appears to have an advantage over D2 agonists and earlier full D1 agonists such as ABT-431, which were often limited by severe cardiovascular hypotension (Bezard et al., 2024; Kaye et al., 2025). Tavapadon produces consistent, modest decreases in systolic and diastolic blood pressure, but these changes are generally mild and have not translated into a significant increase in hypotension-related clinical events such as syncope (Fernandez et al., 2026a; Pringsheim et al., 2021).

A major concern with traditional D2/D3 agonists is the substantial incidence of impulse-control disorders, such as pathological gambling or hypersexuality, occurring in up to 17% of patients (Bezard et al., 2024; Kawahata et al., 2024). Tavapadon, in contrast, has shown no excess signal of impulse-control disorders relative to placebo during the available 26–27-week controlled trial period (Fernandez et al., 2026a), consistent with its low D3 receptor affinity (Ki of at least 6720 nM), given that mesolimbic D3 overstimulation is mechanistically linked to reward-processing dysregulation (Voon et al., 2011; Weintraub et al., 2010). Regarding cardiac safety, phase 1 single-ascending-dose studies initially noted a small, dose-related increase in mean QTcF interval (up to 11.7 ms) (Riesenberg et al., 2020); however, multiple-ascending-dose studies and large-scale phase 3 trials found no clinically significant QTc prolongation or electrocardiogram abnormalities, even at higher doses. Although a dose-dependent QTc trend was observed in single-dose studies, thorough QT studies and 26-week phase 3 data confirmed that the upper bound of the 90% confidence interval remained below the 10 ms regulatory threshold of concern, indicating no clinically actionable repolarization risk (Pahwa et al., 2026; Riesenberg et al., 2020).

## Potential clinical positioning

In early PD monotherapy, tavapadon has demonstrated efficacy versus placebo and may be considered a candidate alternative to levodopa and D2 agonists, a positioning that remains hypothesis-generating in the absence of head-to-head trials (Kaye et al., 2025). Levodopa remains the gold-standard therapy for motor symptoms but carries a substantial long-term dyskinesia burden; current American Academy of Neurology guidance suggests dopamine agonists may be preferred in patients under 60 years to delay levodopa-induced complications (Kawahata et al., 2024). Phase 3 data from TEMPO-1 show that fixed-dose tavapadon significantly improves MDS-UPDRS motor scores compared with placebo, with an acceptable safety profile (Pahwa et al., 2026).

For fluctuating PD, tavapadon may serve as an adjunctive therapy. TEMPO-3 demonstrated that adding once-daily tavapadon to a stable levodopa regimen increased good ON time, on-time without troublesome dyskinesia, by 1.1 h and significantly reduced OFF time (Fernandez et al., 2026a; Kaye et al., 2025), which is relevant for patients whose current levodopa regimen no longer provides sustained relief throughout the day (Li et al., 2025).

Based on current data, tavapadon may be preferentially considered for patients with early PD who wish to delay levodopa initiation, particularly those sensitive to the somnolence or cognitive dulling associated with D2/D3 agonists, and for levodopa-treated patients with motor fluctuations who continue to experience more than 2 h of daily OFF time despite optimized D2 agonist or catechol-O-methyltransferase inhibitor therapy. Its apparent lack of somnolence-inducing effects relative to D2 agonists may make it suitable for patients with active lifestyles or those who must avoid sudden sleep attacks (Kawahata et al., 2024; Kaye et al., 2025), and its proposed effects on cortical D1/D5-mediated cognitive and motivational circuits raise the mechanistic hypothesis, not yet directly tested in tavapadon trials, that it could carry a more favorable cognitive risk profile than anticholinergics or D2 agonists for patients at risk of cognitive decline; this requires direct comparative cognitive-outcome data before being treated as an established advantage (Bezard et al., 2024; Riesenberg et al., 2020). Tavapadon also has predictable drug interactions, is cleared primarily via CYP3A4, and is unaffected by food intake, which may support convenient once-daily dosing and long-term adherence (Kaye et al., 2025; Sohur et al., 2018).

Tavapadon remains unproven, however, in several real-world subgroups excluded from trials, including patients with pre-existing psychosis, moderate-to-severe cognitive impairment (Montreal Cognitive Assessment score below 21), or significant orthostatic hypotension (systolic drop greater than 20 mmHg). Cautious use, or avoidance, in these populations is advisable until dedicated trials are performed.

## Future directions

Tavapadon has accumulated enough clinical evidence to be considered a notable new drug candidate for Parkinson’s disease, though several questions remain. Chief among them is that the pivotal trials to date have compared tavapadon with placebo rather than with the drugs used in routine clinical practice. In early PD, fixed-dose tavapadon improved combined MDS-UPDRS Part II and III scores over 26 weeks in TEMPO-1, and in patients already receiving levodopa with motor fluctuations, TEMPO-3 showed improvement in good ON time and reduction in OFF time. These findings are encouraging but do not establish that tavapadon is superior to levodopa, extended-release levodopa, or older dopamine agonists such as pramipexole, ropinirole, or rotigotine (Fernandez et al., 2026a; Pahwa et al., 2026).

Head-to-head trials comparing tavapadon with D2/D3 dopamine agonists would be a useful next step, given that tavapadon acts primarily through the D1/D5 pathway whereas many older agonists predominantly stimulate D2/D3 receptors. This distinction could plausibly reduce adverse effects associated with D2/D3 stimulation, such as impulse-control disorders, hallucinations, somnolence, and edema, though this remains a hypothesis that placebo-controlled trials cannot resolve (Bezard et al., 2024; Riesenberg et al., 2020). A comparison with levodopa-based strategies would also be informative: levodopa remains the most effective symptomatic therapy but is often limited over time by wearing-off and dyskinesia, and whether tavapadon can delay the need for higher levodopa doses or reduce OFF time without troublesome dyskinesia requires direct testing. Future trials should assess motor scores, time to levodopa initiation, changes in levodopa dose, dyskinesia-free ON time, quality of life, falls, sleepiness, and treatment discontinuation (Fernandez et al., 2026a; Pahwa et al., 2026).

Long-term safety also warrants further study. The phase 3 trials were long enough to demonstrate short-term benefit, but PD treatment typically continues for years, and some dopaminergic adverse effects, particularly impulse-control problems, hallucinations, sleep attacks, and dyskinesia, may take longer to emerge. Trial populations to date have not been fully representative of patients seen in routine practice, and future studies should include older adults, people with mild cognitive impairment, patients with autonomic symptoms, and more racially diverse groups (Fernandez et al., 2026a; Pahwa et al., 2026).

Specific priorities for future research include a delayed-start trial comparing tavapadon with placebo over 12 to 18 months, followed by a washout phase, to distinguish symptomatic effect from potential disease-modifying activity; head-to-head non-inferiority trials against pramipexole in early PD and against opicapone or entacapone as adjuncts, to establish comparative effectiveness on good ON time and tolerability; and longitudinal cognitive monitoring using the MDS-UPDRS Part I and comprehensive neuropsychological batteries to test the theoretical cognitive-safety advantage of D1/D5 over D2/D3 agonism.

At present, tavapadon should be viewed as a symptomatic treatment rather than a disease-modifying therapy. Improvement in MDS-UPDRS scores indicates symptomatic benefit but does not establish an effect on the underlying neurodegenerative process, and past PD trials illustrate how difficult disease modification is to demonstrate: delayed-start trials of levodopa and pramipexole, for instance, did not definitively show that earlier treatment altered the long-term disease course (Schapira et al., 2013; Verschuur et al., 2019). Testing for a disease-modifying effect of tavapadon would require a more robust design than a simple placebo-controlled symptomatic trial, potentially incorporating delayed-start designs, withdrawal phases, longer follow-up, and biomarkers such as dopamine transporter imaging, alpha-synuclein seed amplification assays, neurofilament light chain, and wearable sensor data. Even a delayed-start or withdrawal design has important interpretational limits: symptomatic differences can persist through a washout period because of pharmacodynamic carryover or motor reserve rather than disease modification, and biomarker changes would be complementary, not definitive, evidence. Taken together, these approaches, while imperfect, may help distinguish a change in disease biology from a purely symptomatic effect, but current available data do not support any claim of neuroprotection or disease modification for tavapadon (Sun et al., 2024).

There is also interest in whether D1/D5-based therapy might have applications beyond Parkinson’s disease. One possibility is dementia with Lewy bodies, which combines parkinsonism with cognitive symptoms in patients who are particularly sensitive to dopaminergic side effects such as hallucinations and confusion; mevidalen, another D1-pathway drug, has shown some improvement in motor function without significant worsening of non-motor symptoms in this population, though this cannot be assumed to generalize to tavapadon (Biglan et al., 2022). Huntington’s disease presents a more complicated possibility, since dopaminergic signaling changes over its course, with hyperkinetic movements more prominent early and rigidity and parkinsonian features more prominent later; D1/D5 stimulation might aggravate chorea in some patients, so any future study would likely need to focus on specific subgroups rather than the full Huntington’s disease population (Chen et al., 2013). Restless legs syndrome appears to be a less promising future indication: dopamine agonists were once standard treatment but have fallen out of favor because of augmentation, in which symptoms start earlier in the day, worsen, or spread to other body parts, and recent mechanistic work suggests D1-related pathways may contribute to this exacerbation during chronic dopaminergic treatment, making tavapadon an unlikely candidate for this indication at present (Ferré et al., 2026; Winkelman and Wipper, 2026). Future research, in short, needs to move past placebo comparisons: direct comparisons with existing therapies, longer follow-up, broader patient populations, and biomarker or digital measures of long-term effect would all sharpen the picture.

## Conclusion

Tavapadon offers a pharmacologically distinct approach to dopaminergic stimulation in Parkinson’s disease. Phase 1 and phase 2 studies found PF-06649751/tavapadon to be well tolerated and efficacious as an oral, once-daily therapy that improved motor symptoms in early PD, and subsequent phase 3 studies have provided further evidence that tavapadon can improve motor function as monotherapy in early PD and reduce OFF time when combined with levodopa in patients with motor fluctuations (Fernandez et al., 2026a; Pahwa et al., 2026; Riesenberg et al., 2020; Sohur et al., 2018). Its principal advantage lies in its receptor selectivity: as a D1/D5 partial agonist, it may offer motor benefit while circumventing some of the limitations of older D2/D3 dopamine agonists. The pivotal randomized, placebo-controlled efficacy evidence remains largely short-term (26–27 weeks) and derived from trials with restrictive entry criteria; the 58-week open-label TEMPO-4 extension adds preliminary evidence on durability and levodopa-sparing potential but, lacking randomization and a control arm, cannot establish long-term safety or superiority. It remains premature to conclude that tavapadon is safer or more effective than existing treatments; direct comparative studies and longer-term safety data are still needed.

For now, tavapadon should be regarded as a promising symptomatic therapy rather than a disease-modifying one. It may prove useful in early PD, in patients seeking to postpone or reduce levodopa exposure, or as adjunctive treatment for patients with OFF periods. Its ultimate place in therapy will depend on further data regarding long-term tolerability, dyskinesia risk, neuropsychiatric safety, and real-world adherence. If ongoing and future head-to-head trials confirm durable efficacy and long-term safety, tavapadon may become a valuable addition to the PD treatment armamentarium; until then, it remains an investigational agent with a strong biological rationale.
